# The Influence of Racial Differences in the Demand for Healthcare in South Africa: A Case of Public Healthcare

**DOI:** 10.3390/ijerph17145043

**Published:** 2020-07-14

**Authors:** David Mhlanga, Rufaro Garidzirai

**Affiliations:** 1 Department of Accountancy, University of Johannesburg, Auckland Park, Johannesburg 2092, South Africa; 2 Department of Management, Walter Sisulu University, Butterworth 4960, South Africa; garidzirairufaro@gmail.com

**Keywords:** demand, public healthcare, racial differences, South Africa

## Abstract

The study sought to analyse the influence of racial differences in the demand for public healthcare in South Africa, using the 2018 General Household Survey (GHS) data. This was completed to understand if race still plays a role in access to healthcare in post-apartheid South Africa. Logistic regression analysis revealed that race significantly explained the variance in demand for public healthcare, with White populations having the lowest probability of demand for public healthcare compared to other races. Consequently, the study noted that racial differences still play a critical role in affording one access to healthcare after assessing the situation obtaining in public healthcare. Therefore, the study recommends that the government of South Africa should create policies that encourage equal access to basic services in addressing racial inequality in the country.

## 1. Introduction

Race and socioeconomic status (SES) were important determinants of the utilisation of and access to healthcare services during the apartheid era in South Africa [1,2]. South Africa is now 25 years into democracy, but inequality remains high in the country in many sectors, and the health sector is not an exception [3]. The ability to have access to quality services is mainly influenced by the socio-economic status of the people, rather than the need for care [1]. In South Africa, the majority of people depend on public healthcare facilities to access healthcare services. In 2019, Statistics South Africa (StatsSA) reported that 71.5% of households used public healthcare facilities whenever they needed care, while 27.1% of households used private healthcare facilities when they required healthcare [4]. The statistics further showed that 0.7% of households consulted traditional healthcare facilities, such as traditional healers, when they were ill [4]. In 2017, the Department of Health (DoH) revealed that the private sector spent 4.4% of gross domestic product (GDP) on health, but only provided healthcare to 16% of the population, while the public sector spent 4.1% of GDP on health, but had to provide care to 84% of the population [5,6].

Moreover, the 2016 General Household Survey (GHS) indicated that only 17 in every 100 South Africans have access to medical insurance [2]. To exacerbate matters, almost 45 million or 82 in every 100 South Africans, have no medical aid at all. All these people depend on public healthcare facilities [2]. Similarly, the 2018 GHS (Table 1) highlighted that only 9.9% of Black African households had access to medical insurance, while Indians and Coloureds had 52% and 17.1%, respectively [4].

The number of Whites with medical insurance was 72.9%, with a 16.4% health insurance coverage nationally, as shown in Table 1. The other reality is that the number of people who depend on public healthcare is much higher since undocumented foreign nationals also depend on public healthcare to access healthcare services [4]. High demand for public healthcare facilities has an impact on “the timeliness, range and quality of services provided to users” [1]. Another reality in South Africa is that private healthcare is generally administered through medical aid schemes which are excessively costly for the reach of many. For this reason, more households depend on public healthcare facilities than the costly private healthcare facilities.

Young [7] stated that although public healthcare is government funded, the sector suffers from many challenges compared to private healthcare. It is believed that many of the problems facing the public healthcare in South Africa originated from the apartheid era (1948–1993) where the healthcare system was fragmented with discriminatory effects among the racial groups of Blacks, Coloureds, (mixed race), Indians and White [8]. Young [7] further argues that public healthcare is characterised by long waiting times, “long wait times, rushed appointments, old facilities, and poor disease control and prevention practices”, compared to private healthcare that has opposite features which include “short wait times, appointments are not rushed, better facilities, and proper disease control and prevention practices”. Maphumulo and Bhengu [8] concur with Young [7] by contending that the quality of healthcare in South Africa, especially in the public healthcare sector, deteriorated over the years. Maphumulo and Bhengu [8] posit that public healthcare is characterised by “prolonged waiting time because of shortage of human resources, adverse events, poor hygiene and poor infection control measures, increased litigation because of avoidable errors, shortage of resources in medicine and equipment and poor record-keeping”. These findings are supported by the high morbidity and mortality rates. StatsSA [9] reports that 60 out of 2000 infants die every year at birth, while 600 out of 2000 adults die every year in South Africa. StatsSA [9] further reports that the morbidity level in South Africa is significantly high and is dominated by tuberculosis, influenza, human immunodeficiency virus, diabetes and heart diseases.

Motivated by these revelations and scarcity of empirical evidence in South Africa on this subject [10,11], the study investigated the influence of race in demand for healthcare, taking public healthcare as a case study. The study was premised on the reality that, in South Africa before democracy, there was unequal access to services, which had been institutionalised by the apartheid rules and regulations that discriminated against the natives who were the majority during that time. This study is important in influencing the development of a policy that addresses inequality in the country.

## 2. Literature Review

### 2.1. Theoretical Background

Demand is defined as a concept of economics that describes the consumer’s desire to pay a price for goods and services [12]. Holding all other factors constant, when the price of a good or service rises, demand will decline, whereas a decrease in the price of a good or service will increase demand [13]. According to Grossman [14] the demand for healthcare is derived from the need for health services. Healthcare is demanded as a means for consumers to achieve a larger stock of health capital. The demand for healthcare is unlike most other goods since individuals allocate resources to consume and produce health [14]. In this study, use of healthcare and demand for health are used interchangeably.

The link between race and the demand for public healthcare can be illustrated by the Grossman model [14]. The theory postulates that people demand healthcare services despite race, age and affordability. Thus, there is no race that is immune to diseases, making the demand for healthcare unavoidable. People demand healthcare to obtain total satisfaction from consuming the services. However, the distinguishing factor between race and the demand for public healthcare is the quality of healthcare. For instance, there is a certain race that is more inclined to the use of private healthcare, while other races demand public healthcare. This notion is also linked to the income hypothesis theory that suggests that rich people demand private healthcare, while poor people demand public healthcare [15]. Simply put, it is difficult for a poor person to utilise private healthcare when they can barely afford basic goods and services, such as food, shelter and clothing. Consequently, by electing to forgo private healthcare, an opportunity cost is incurred when one chooses food and shelter consumption. In most cases, Blacks experience that, especially in developing countries [16]. As a result, the majority of Black people derive total satisfaction from public healthcare, while Whites and Indians derive their satisfaction from private healthcare [17]. The Public Policy Initiative [18] emphasises the fact that the majority of Whites have more access to medical insurance since they visit private hospitals, while the majority of Blacks have access to public healthcare.

### 2.2. Empirical Literature Review

#### 2.2.1. Public Health and Race

The relationship between the demand for public healthcare and race can be traced back to the 1990s [19,20]. In 1992, Hahn and Lefkowitz examined the association between the use of public healthcare and race, using multivariate analysis. The authors found that race was one of the major determinants of the demand for healthcare. A few years later, Freiman [20] studied the use of public healthcare by different races in Europe. The results of the study found that Blacks and Hispanics showed greater demand for public medical care compared to Whites. David et al. [21] also support the findings of [19,20]. The authors confirmed that Whites and Indians preferred the use of private healthcare instead of public healthcare. The above-mentioned researchers unanimously concurred that the Black ethnic group could not afford private healthcare. Thus, there was an excessive demand for public healthcare among the Black populace.

Recent studies on race and the use of public healthcare seem to support the early 1900’s findings [19,22]. Chase et al. [22] and Stepanikova and Oates [23] compared the use of public healthcare by Blacks, Hispanics, Whites and Spaniards. Both of these studies employed logistic regressions and found that the Black and Hispanic population groups made use of public hospitals as opposed to and Spaniards. Villagra et al. [24] also carried out the same study and found different results. Interestingly, the authors found that Spaniards made use of public healthcare more than the other races.

#### 2.2.2. Public Health and Gender

In their survey, Moran and Valle [25] concluded that females were more vulnerable to diseases than men. This notion was supported by several researchers such as [26,27,28]. Perelman et al. [28] examined the use of healthcare by females and males in Portugal, using multivariate regressions. The results of the study revealed that females were likely to experience more chronic diseases, thereby require more healthcare than males. A study done on 138 countries investigated the relationship between the use of public healthcare by females and males and found that females made use of public hospitals more often than men [29]. The authors further found that females’ chronic diseases emanated from childbirth and childcare. Dunga [10] further argued that a woman’s need for public healthcare is unavoidable since they face many reproductive challenges. Using a multiple logistic regression model, the authors of [30] examined the association between gender and healthcare in Vietnam. The authors concluded that gender was one of the most influential factors in health. Amongst the many reasons why females demanded more public healthcare was the need for contraceptives, regular check-ups, and child delivery.

#### 2.2.3. Public Health and Age

The association between the demand for healthcare and age remains unclear since empirical literature shows mixed results. For instance, Melzer [31] observes that age is a factor that influences the demand for public healthcare but is not that important. Instead, age and affordability are positively related. The author found that both old and young people who are financially stable make use of medical aid services and private healthcare. Zhang et al. [32] conducted a similar study but found different results from Melzer [31]. The authors found that young people made use of public healthcare compared to old people. A recent study by Park and Hong [33] found contrary results. The authors examined the association between public healthcare and age (20–39 years and 40–64 years) in Korea and found that younger adults required public healthcare more than older adults. Rehnberg [34] found no significant relationship between public healthcare and age. The author attached the health status to age, instead. The author argued that a positive relationship exists between health status and age. Thus, age has a positive effect on the health status of an individual.

#### 2.2.4. Public Health and Household Size

Morudu and Kollamparambil [11] argue that there is a positive relationship between the use of public healthcare and household size in South Africa. Their study found that South Africa’s population was on a surge, and the majority was vulnerable to poverty and the lack of medical insurance. Thus, the greater the household size, the greater the use of healthcare. These findings were similar to those of Kirigia et al. [35], who observed a negative relationship between health insurance and household size. Thus, households without medical aid were likely to visit public hospitals. Furthermore, Lofti et al. [36] investigated the factors that influenced the demand for healthcare in Iran. The study found that an inverse relationship between household size and the demand for private healthcare existed. Contrarily, Govender [37] argued that household size did not matter, provided the household head could afford to transport the dependents to the public hospital. In this instance, household size was likely to demand more public care. In the same vein, the lower the household size, the less the demand for public healthcare.

#### 2.2.5. Public Health and Homeownership

There are several discussions on whether owning a house influenced the demand for public healthcare. Guided by the absolute income theory, one can argue that the demand for healthcare is mainly influenced by whether a person owns a house or not [15,38]. Kawachi and Kennedy [15] argue that the ability to own a house increases the chances of an individual’s demand for private healthcare. Thus, individuals can also afford basic commodities such as food, shelter and health insurance. Boz et al. [38] examined the demand for public health factors in Edirne city and found that gender, household size and the acquisition of assets were the main determinants of demand for healthcare. The authors also observed that households that owned houses were more likely to demand private healthcare than public healthcare.

From the empirical literature discussed above, it seems there is no consensus on how age, gender, race, homeownership and household size influenced the demand for public healthcare. Most of the literature shows that in developing countries, females, Blacks, small household size, and old age demand public healthcare, while the minority states otherwise. Mixed results provide leeway for researchers to contribute to the current debate. This is achieved using the logit regression model, discussed in the subsequent section.

## 3. Research Methodology and Data Description

The study used data from the 2018 GHS of South Africa. Relevant data were extracted from the data set to fulfil the objective of the study.

### 3.1. Dependent Variable

The dependent variable is dichotomous, that is, the household either chooses public healthcare or private healthcare. This is represented by a 0 or 1, respectively. The dependent variable was generated from the question which asked participants on which medical healthcare institution they would go to first when they are ill or when they are involved in an accident (public, private, traditional). The data were recorded to appear in a binary form wherein the public healthcare institution choice was named 1, while other healthcare institutions took the 0. The subsequent section discusses the independent variables used in this study.

### 3.2. Independent Variables

Guided by the works of Andersen’s Behavioural Model [39], Grossman’s Model of Demand for Health [10], and the literature on the determinants of demand for healthcare facilities, the following independent variables were used in the study:

Race/Population Group. Is a categorical variable which explains the grouping of humans based on shared physical or social qualities? In this variable 1 = African Black, 2 = Coloured (mixed race-people of mixed White and Black African or Asian ancestry), 3 = Indian (Southeast Asians), 4 = White. The variable is expected to be negative or positive, depending on the reference category in the dummy variable.

Gender. This variable is a dummy variable where 1 = male and 0 other. The variable is expected to be positively influenced by the choice of public healthcare centres for women and negative for private healthcare institutions.

Age. This variable is a continuous variable which explains the number of years of an individual. The variable is expected to be a positive influence on demand for public and private health facilities.

Household size. This explains the number of people in the household. The variable is expected to have a positive influence on the choice of public institutions and traditional institutions, and a negative on the private institution.

Own house. The variable is a dummy variable where 1 denoted owning a house, while 0 was otherwise. The variable can have a negative or positive influence on the choice of a healthcare provider.

### 3.3. Empirical Model: The Logit Model

The nature of the dependent variable necessitated the use of the logit model. The equation of the logit model transforms the log-odds of success to a linear component as shown below:

(1) $$\log\left(\frac{\pi_{i}}{1-\pi_{i}}\right)=\overset{K}{\underset{k=0}{\sum }}x_{ik}\beta_{k}\;i=1,2,\ldots ,N$$

In Equation (1), using the maximum likelihood estimation implies finding parameters where the probability of the observed data is greatest. To estimate the logit model successfully, we should first apply it to the probability that Y=1, also written as $\hat{P}$. The probability that Y=0 is written as $1-\hat{P}$. Y=1 and Y=0 indicate whether the household demand public healthcare or not, respectively. This will drive us to the following equation:

(2) $$\ln\left(\frac{P}{1-P}\right)=\beta_{0}+\beta_{1}X$$

The expected probability that *Y* = 1 for all the values of *X* is calculated as shown below:

(3) $$\hat{P}=\frac{\exp\left(\beta_{0}+\beta_{1}X\right)}{1+exp\left(\beta_{0}+\beta_{1}X\right)}=\frac{e^{\beta_{0}+\beta_{1}X}}{1+e^{\beta_{0}+\beta_{1}X}}$$

The model with the variables used as the determinants of demand public healthcare will be expressed as:

(4) $$\ln\left(\frac{P}{1-P}\right)=\beta_{0}+\overset{n}{\underset{i}{\sum }}\emptyset_{i}+\overset{n}{\underset{j}{\sum }}\emptyset_{j}+\varepsilon$$

In Equation (4), $\sum_{i}^{n}\emptyset_{i}$ shows all the factors in the model, while all the covariates are represented by $\sum_{j}^{n}\emptyset_{j}$. Substitution of the above equation with Z will make the equation appear as follows:

(5) $$Z=\beta_{0}+\emptyset_{1}Race+\emptyset_{2}Age+\emptyset_{3}Household\;size+\emptyset_{3}House\;ownership+\emptyset_{4}Gender+\varepsilon$$

## 4. Results and Discussion

Table 2 shows the study’s population distribution. The study used a sample of 20,908 across all the South African provinces. The study targeted all the racial groups in South Africa, namely, Black African, Coloured, Indian and White. Table 2 shows 17,361 (80.9%) Blacks constituted the remaining 19%. This remaining 19.1% was constituted as follows: Whites (9.5%), Coloureds (7.1%) and Indian (2.4%). Statistics [4] further highlight that the sample reflected the structure of the South African population.

Table 3 depicts the gender of the population under study. The results show that 11,948 (58.4%) were male-headed households, while 8960 (41.6%) were female-headed households. Thus, the sample size of the study had more male-headed households than female-headed households. In the same table, the descriptive statistics show a total number of 20,908 households. Noteworthy is that all the households are representative of all the provinces in South Africa. Out of 20,908 households, 15,716 (which is more than 70%) made use of public clinics or hospitals when they were ill or involved in an accident, while 4971 (which is also more than 24%) visited private healthcare facilities when they fell ill or were involved in an accident. Only 166 (close to 1%) visited traditional health facilities.

### Regression Results

This section discusses the logistic regression results depicted in Table 4. Of significant note is that the model used was a good fit, as the omnibus test illustrated a probability value of 0.000. Thus, the dependent variable (use of public healthcare) was identified as 1.

The results in Table 2 illustrate that race was a significant variable in influencing the demand for public healthcare in South Africa after taking Black as a reference category. The results indicated that being White had a negative significant influence on the demand for public healthcare in South Africa with a *p*-value of 0.000 and an odds ratio of 0.037. The demand for public healthcare for White households was 0.037 lower compared to all other three races combined. These findings are in line with the findings of Chase et al. [23] who concluded that Whites in South Africa demand private healthcare more than all other races in South Africa. The results also showed that being Indian was negatively significant in influencing, with the probability of demand for public healthcare in South Africa with a *p*-value of 0.000 and an odds ratio of 0.119. Indians had a lower probability of using public healthcare compared to Coloureds and Blacks. The probability of demand for public healthcare was 0.119 lower for Indians compared to Coloureds and the Black population. This finding was also arrived at by David et al. [21], who found in their study that racial differences ultimately affect the demand for healthcare.

Being Coloured was also significant in influencing the probability of demand for public healthcare. Although the variable had a negative influence, the influence was not as strong as that of Indians and Whites, with a coefficient of −0.846. The variable Coloured was significant with a p-value of 0.000 and an odds ratio of 0.429. Coloureds had a lower probability of demanding public healthcare by 0.429 compared to the Black population in the reference category, with 2.734. Freiman [20] also found that Coloureds demand private healthcare more than public healthcare. The Black population group, which was the reference category, was the group with the highest probability of demand for public healthcare. Noteworthy, being Black increased the use of public healthcare in South Africa. Being Black had a positive influence on the probability of demand for public healthcare. Therefore, in South Africa, the demand for public healthcare is still influenced by the population group of the individual. These results are supported by the authors of [23,24], who also found that Blacks demanded public healthcare most. This was in line with the findings of the GHS data which indicated that only 9.9% of Black African households had access to healthcare through medical insurance, while Indians and Coloureds had 52% and 17.1%, respectively. The data also indicated that 72.9% of the White population could access healthcare through medical insurance, while nationally only 16.4% of the population could have access to healthcare through medical insurance.

The results also showed that the age of household head, household size, house ownership and gender were also significant in influencing the demand for healthcare in South Africa. Gender of the household head was significant, with a positive influence on the demand for public healthcare of a *p*-value of 0.000 and an odds ratio of 1.639. The meaning of the variable is that being female increased the probability of demand for public healthcare compared to males. These results were in line with the findings of the authors of [10,30], who observed that women were likely to frequent health facilities more often than their male counterparts. This could be true in the case of public healthcare facilities, as the general household data show that from a total of 20,908 households interviewed, 15,716 (71.5%) households visited public health institutions compared to 4971 (27.1%) who visited private healthcare facilities.

Moreover, the variable house ownership was significant in influencing the demand for use of public healthcare facilities. The results indicated that owning a house in South Africa reduced the probability of choosing public healthcare compared to households without homeownership. The variable was significant at 1% level of significance, with a *p*-value of 0.000, while the odds ratio was 0.699. The same findings were made by Boz et al. [38]. The results further indicate that age positively influences the demand for public healthcare. The variable was significant at 1% level of significance, with a *p*-value of 0.000 and an odds ratio of 1.003. The results showed that a 1% increase in the age of an individual led to an increase in the demand for public healthcare by approximately 1.003. Dunga [10] reports that an increase in the demand for public healthcare is a result of an increase in the population in South Africa. The majority of the population is living under the poverty line of R 992 per month.

The results also indicated that household size was a significant variable that influenced the probability of demand for public health facilities. The variable had a positive influence on the demand for public health facilities and it was significant at 1% level of significance, with a *p*-value of 0.000 and an odds ratio of 1.109. The meaning of the results was that the size of the household increased the probability of demand for public healthcare. A rise in the size of the household by one unit increased the probability of demand for public healthcare by 1.1090. This could be associated with the notion that an increase in the size of the household sometimes puts a lot of pressure on the amount of income the household earns, which in turn reduces the chances of the household demanding private healthcare due to low levels of income and poverty. The report by Morudu and Kollamparambil [11] also revealed that groups most affected by poverty in South Africa were Black South Africans, the unemployed, the less educated, female-headed households, large families and children.

## 5. Conclusions and Policy Recommendations

It is the right of every citizen to have access to healthcare. This is in line with the Sustainable Development Goal number 3 that safeguards the health and protection of every citizen and endorses happiness for all. Good health forms the basis of a good society. Thus, it is of paramount importance for the government to provide efficient and non-discriminatory services. Therefore, this study aimed to analyse the influence of racial differences in access to public healthcare in South Africa. The study also considered other variables such as gender, age, house ownership and household size. The results of the study showed that the majority of South Africans made use of public healthcare. Among the statistically significant variables was race. Thus, the Black population made more use of public healthcare compared to Whites, Indians and Coloureds. It seems the majority of the Black population could not afford private healthcare. This is why the Black population demanded public healthcare. The study also found that older people made use of public healthcare as they were more susceptible to diseases than the young. Furthermore, females, house owners and household size were statistically significant in explaining the demand for public healthcare. Based on the study’s results, the study recommends that the government find ways to promote the Black population in terms of employment so that they can also afford private healthcare. Alternatively, the government should invest more in public healthcare to improve the efficiency and quality of the services rendered.

### Limitations of the Study

Though the study achieved its objective, it has its limitations. The study is confined to South Africa and other important variables such as education level were not included due to data limitations. Therefore, extending the study to a regional level and including other important variables will be a more and rigorous academic undertaking.

## Figures and Tables

**Table 1:** Access to private healthcare through health insurance.

Access to Private Healthcare through Health Insurance				
Black African	Coloured	Indian/Asian	White	South Africa
9.9%	17.1%	52%	72.9%	16.4%

Source: Author’s calculations of GHS data.

**Table 2:** Study of population distribution.

Race	Count out of 20,908	Percentage
Blacks African	17,361	80.9%
Coloured	1659	7.1%
Indian	391	2.4%
White	1497	9.5%

Source: Author’s calculations of GHS data.

**Table 3:** Study’s gender distribution and use of healthcare facilities.

Study’s Gender Distribution		
Gender	Frequency	Percentage
Male-headed households	11,948	58.4%
Female-headed households	8960	41.6%
Total	20,908	100%
**Use of healthcare facilities**		
Public healthcare	15,716	75%
Private healthcare	4971	24%
Traditional healthcare	166	0.8%
Missing	55	0.2%
Total	20,908	100

Source: Author’s calculations of GHS data.

**Table 4:** Logistic regression results.

Variable	B	S.E	Wald	Df	Sig.	Exp(B)
Race			2025.8	3	0	
Coloured	−0.846	0.059	208.759	1	0	0.429
Indian	−2.127	0.11	370.972	1	0	0.119
White	−3.297	0.081	1658.103	1	0	0.037
Age of household	0.003	0.001	6.617	1	0	1.003
Household size	0.104	0.009	120.774	1	0	1.109
House ownership	−0.358	0.04	78.567	1	0	0.699
Gender/female	0.494	0.039	159.909	1	0	1.639
Constant	1.006	0.075	179.677	1	0	2.734

Model Summary Step: Two-log likelihood = 19,055.728a; Cox and Snell R square = 0.175; Nagelkerke R square = 0.260. Omnibus tests of model coefficients: chi-square step = 3962.467; block = 3962.467; model = 3962.467; df 7 Sig = 0.000. Source: Author’s manipulation.

## References

[1] Lalloo R., Smith M.J., Myburgh N.G., Solanki G.C. (2004). Access to health care in South Africa-the influence of race and class. S. Afr. Med. J..

[2] Burger R., Christian C. (2020). Access to health care in post-apartheid South Africa: Availability, affordability, acceptability. Health Econ. Pol. Law.

[3] Sulla V., Zikhali P. (2018). Overcoming Poverty and Inequality in South Africa, An Assessment of Drivers, Constraints and Opportunities.

[4] Statistics South Africa (StatsSA) (2018). General Household Survey.

[5] Department of Health (DoH) (2018). Medical Schemes Act of South Africa Amendment Bill, South Africa.

[6] Institute of Economic Justice (2019). Fact Sheet: Funding The Right To Health.

[7] Young M. (2016). Private vs. Public Healthcare in South Africa. https://scholarworks.wmich.edu/cgi/viewcontent.cgi?article=3752&context=honors_theses.

[8] Maphumulo W.T., Bhengu B.R. (2019). Challenges of quality improvement in the healthcare of South Africa post-apartheid: A critical review. Curationis.

[9] Statistics South Africa (2020). Mortality Rate. http://www.statssa.gov.za/?s=mortality.

[10] Dunga S.H. (2019). Analysis of the Demand for Private Healthcare in South Africa. Stud. Univ. Babeș-Bolyai Oeconomica Sciendo.

[11] Morudu P., Kollamparambil U. (2020). Health shocks, medical insurance and household vulnerability: Evidence from South Africa. PLoS ONE.

[12] Mohr P., Fourie L. (2008). Economics for South African Students.

[13] Babalola O. (2017). Consumers and their demand for healthcare. J. Health Med. Econ..

[14] Grossman M. (1972). On the concept of health capital and the demand for health. J. Polit. Econ..

[15] Kawachi I., Kennedy B.P. (2002). The Health of Nations.

[16] Hartwig J., Sturm J. (2018). Testing the Grossman model of medical spending determinants with macroeconomic panel data. Eur. J. Health Econ..

[17] Shafrin J., Green B.A., Richard M., Katie E., Kwanza P. (2019). Quantifying the Economic Burden of Myelodysplastic Syndromes Among Elderly US Patients. Myeloid Dis..

[18] Public Policy Initiative (2015). Precision Medicine: The Future of Healthcare?.

[19] Hahn B., Lefkowitz J. (1992). Annual Expenses and Sources of Payment for Health Care Services. National Medical Expenditure Survey Research Findings.

[20] Freiman M.P. (1998). The demand for healthcare among racial/ethnic subpopulations. Health Serv. Res..

[21] David R., Williams M.P.H., Harold W., James S.J. (2003). Racial/Ethnic Discrimination and Health: Findings from Community Studies. Am. J. Public Health.

[22] Chase J.A.D., Russell D., Huang L., Hanlon A., O’Connor M., Bowles K.H. (2020). Relationships between Race/Ethnicity and Health Care Utilization Among Older Post-Acute Home Health Care Patients. J. Appl. Gerontol..

[23] Stepanikova I., Oates G.R. (2017). Perceived Discrimination and Privilege in Health Care: The Role of Socioeconomic Status and Race. Am. J. Prev. Med..

[24] Villagra V.G., Bhuva B., Coman E., Smith D.O., Fifield J. (2019). Health insurance literacy: Disparities by race, ethnicity, and language preference. Am. J. Manag. Care.

[25] Moran K.R., Valle S.Y. (2016). A Meta-Analysis of the Association between Gender and Protective Behaviors in Response to Respiratory Epidemics and Pandemics. PLoS ONE.

[26] Rodríguez-Díaz C.E. (2018). Maria in Puerto Rico: Natural disaster in a colonial archipelago. Am. J. Public Health.

[27] Garenne M. (2015). Demographic evidence of sex differences in vulnerability to infectious diseases. J. Infect. Dis..

[28] Perelman J., Fernandes A., Mateus C. (2012). Gender disparities in health and healthcare: Results from the Portuguese National Health Interview Survey. Cad. Saúde Pública.

[29] Brinda E.M., Rajkumar A.P., Enemark U. (2015). Association between gender inequality index and child mortality rates: A cross-national study of 138 countries. BMC Public Health.

[30] Hui C.K., Xu B., Chung K.F., Partridge M.R. (2018). Green respiratory health care: Time for us all to. Respirology.

[31] Melzer D., McWilliams B., Brayne C., Johnson T., Bond J. (2000). Socioeconomic status and the expectation of disability in old age: Estimates for England. J. Epidemiol. Community Health.

[32] Zhang X., Dupre M.E., Qiu L. (2018). Age and sex differences in the association between access to medical care and health outcomes among older Chinese. BMC Health Serv. Res..

[33] Park S., Hong S. (2020). The association of health care access and utilization with self-perceived health in South Korea: The significance of age. J. Biosoc. Sci..

[34] Rehnberg J. (2020). What Levels the Association Between Income and Mortality in Later Life: Age or Health Decline?. J. Gerontol. Ser..

[35] Kirigia J.M., Seddoh A., Gatwiri D., Muthuri L.H., Seddoh J. (2017). E-health: Determinants, opportunities, challenges and the way forward for countries in the WHO African Region. BMC Public Health.

[36] Lotfi F., Nouraei M.S., Mahdavi G., Keshavarz K., Hadian M. (2017). Factors Affecting the Utilization of Outpatient Health Services and Importance of Health Insurance. Shiraz E-Med. J..

[37] Govender I., Mabuza L.H., Ogunbanjo G.A., Mash B. (2014). African primary care research: Performing surveys using questionnaires. Afr. J. Prim. Health Care Fam. Med..

[38] Boz C., Gor A., Haydar S., Soyuk S. (2016). The Similarities and Differences Analysis of OECD Countries in Terms of Health System Indicators. ACU Sağlık Bil. Derg..

[39] Aday L.A., Andersen R.M. (1974). A Framework for the Study of Access to Medical Care. Health Serv. Res..

